# Are mobile health applications useful for supporting shared decision making in diagnostic and treatment decisions?

**DOI:** 10.1080/16549716.2017.1332259

**Published:** 2017-08-25

**Authors:** Samira Abbasgholizadeh Rahimi, Matthew Menear, Hubert Robitaille, France Légaré

**Affiliations:** ^a^ Canada Research Chair in Shared Decision Making and Knowledge Translation, Population Health and Practice-Changing Research group, CHU de Québec–Université Laval, Research Centre, Saint-François-d’Assise Hospital, Quebec City, QC, Canada; ^b^ Department of Family Medicine and Emergency Medicine, Faculty of Medicine, Université Laval, Quebec City, QC, Canada

**Keywords:** mHealth for Improved Access and Equity in Health Care, mHealth, mobile applications, patient engagement, decision making, health information

## Abstract

Mobile health (mHealth) applications intended to support shared decision making in diagnostic and treatment decisions are increasingly available. In this paper, we discuss some recent studies on mHealth applications with relevance to shared decision making. We discuss the potential advantages and disadvantages of using mHealth in shared decision making in various contexts, and suggest some directions for future research in this quickly expanding field.

## Background

Most health-related decisions occur in a context of scientific uncertainty, the ‘grey zone’ of decision making [1,2]. The probabilistic nature of the diagnosis and treatment of many health problems makes choosing the best course of action difficult [3]. Evidence drawn from population studies may not be easily applied to specific individuals [4], and evidence that imparts ambiguous results regarding treatment options (i.e. close benefit/risk balance) makes this choice even more difficult [5]. One way for clinicians to better apply research evidence is to actively engage patients in decision making about their health, i.e. to adopt a shared decision making (SDM) approach [6].

SDM is defined as a collaborative process that allows patients and their providers to make healthcare decisions together, taking into account the best scientific evidence available as well as the patient’s values and preferences [7]. There are numerous types of interventions for facilitating SDM that target the healthcare team, the patient, or the public [8]. However, patient decision aids remain the most widely known effective strategy [9].

Patient decision aids are tools that help people become involved in decision making. They help to identify the decision that needs to be made, provide information about the options and outcomes based on the best evidence, and clarify personal values [9]. They are designed to complement, rather than replace, counselling from a health practitioner [9]. In order to help individuals and organizations to use/develop decision aids, a group of researchers, practitioners, and stakeholders from around the world established an internationally approved set of criteria called International Patient Decision Aid Standards (IPDAS) for determining the quality of patient decision aids [10]. Patient decision aids can take the form of leaflets, videos, web-based tools, or grids such as the Option grid [9,11]. They can be used outside the consultation (e.g. to prepare for a discussion with a clinician) or within the consultation to help structure more deliberative and egalitarian conversations between patients and their clinicians [12,13].

Systematic reviews indicate that clients who are more active in making decisions about their health have better health outcomes, better healthcare experiences, and get better value for money [14,15]. However, several barriers interfere with implementation of SDM in clinical practice [16,17]. Among these, time constraints, patient characteristics and self-efficacy, and the nature of the decision accounted for are the most frequently perceived barriers.

The digitalization of healthcare has the potential to save time and money, and enable better physician–patient relationships and personalized treatments based on the specific characteristics of patients [18]. Given the aforementioned barriers to the implementation of SDM in clinical practice, digitalization holds both promise and peril. Recently, there has been increasing emphasis on patient-centric e-health solutions [19]. e-health is generally defined as ‘an emerging field in the intersection of medical informatics, public health and business, referring to health services and information delivered or enhanced through the internet and related technologies’ [20]. One of the most important and common e-health services is mHealth, or mobile health. While no standardized definition has been established, mHealth refers to the use of mobile devices such as smartphones, tablets, patient monitoring devices, personal digital assistants, and other wireless devices for patient self-management, as a complement to physicians’ interventions, and to support SDM [21–23]. mHealth can include the complex functions of mobile phones such as mobile applications, or the most basic ones, such as voice and short messaging services (SMS). In 2011, the World Health Organization estimated the number of worldwide mobile phone subscribers at approximately 5 billion [23]. By 2015 it had increased to more than 7.6 billion [24] and is growing. For example, the number of smartphone users in 2017 is estimated to reach 222.9 million in the US [25]. Half of all smartphone owners in the US gather health information through their phone and 19% use health applications (or ‘apps’). Meanwhile, the number of smartphone users in the Middle East and Africa is estimated to reach 140.9 million in 2017 [25].

mHealth is already being applied and tested in diverse health contexts, such as in maternal and child health, and in programs that seek to reduce the burden of diseases linked to poverty, including HIV/AIDS, malaria, and tuberculosis. It could improve timely access to emergency and general health services and information, as well as help manage patient care, reduce drug shortages at health clinics, and enhance clinical diagnosis and treatment adherence [23]. With the fast-growing use of mobile devices throughout society, mHealth could become a useful tool for overcoming some of the identified barriers to SDM and promoting its widespread adoption. However, political will and appropriate actions from key stakeholders are likely needed for mHealth to fulfil this promise.

Next, we discuss some of the promises and perils of the use of mHealth to support SDM, and outline some future research directions that could facilitate technology-mediated SDM on a broad scale.

## Promises of mHealth in shared decision making

Studies have shown that mHealth can improve SDM opportunities and encourage greater participation in medical decision-making [22]. A recent study evaluated the impact of using new technology (e.g. tablets) on engaging in SDM, and found that using mHealth, patients were highly satisfied with patient–provider interactions [26]. Some features of apps such as fast accessibility, easy-to-follow procedures, or affordability could also influence patients’ satisfaction, though studies are required to further explore these relationships. Another study showed that accurate medical information on smartphone applications empowered patients [22]. Keeping information up to date is possible due to update features on apps that automatically integrate the newest medical evidence.

Mobile applications are more accessible than web-based applications and in high-income countries have been found to increase the efficiency of healthcare providers, save them time, and provide a platform for real-time connectivity with their patients and collaborative decision making [27,28]. Apps can also provide better visualization for patients; this may lead to better understanding. For instance, a recently developed app in dentistry provides patients with easily accessible 3D images of implant procedures. This also helps to prepare patients for giving informed consent [29].

Another advantage of mobile apps supporting SDM is that because they can be accessed using smartphones, patients’ decisions can be supported without having to travel to a healthcare centre. For patients living in hard-to-access areas or developing countries this might be the only decision support they can receive. Finally, apps are also cost-efficient based on some studies [30] and could reduce the burden of paper-based documents for both patients and healthcare providers, reducing the risk of losing important information and also benefiting the environment. Unlike with paper-based decision aids, new features can be routinely added to mHealth apps so that patients can immediately benefit from the latest technologies and information.

## Perils of mHealth in shared decision making

Despite these potential advantages of mHealth for SDM, there are some disadvantages. First, overuse of mHealth apps in the SDM context may undermine the quality of the patient–physician relationship if healthcare providers use them to replace spending time with patients [27]. In addition, in cultures or subcultures where a strong hierarchy of authority makes SDM less likely to take hold in the clinic, a mobile app that challenges the traditional doctor/patient roles is unlikely to change the situation [31].

Mobile applications could also increase health disparities if members of vulnerable populations lack the health literacy skills to take full advantage of the risk/benefit information they offer, lack knowledge of how to use the app, or simply lack access to them [21,32]. In rural areas worldwide, only 67% of the population has mobile network coverage [33]. In low-income countries, in 2016, only one out of seven people were using the Internet and bandwidths are low [33], making apps that depend on the Internet less accessible in these contexts. Some vulnerable populations, such as the elderly or those with low incomes, may not have entered the smartphone culture, may lack the money to buy them, or may not be comfortable with touchscreens [34]. Designing and developing apps along with the most vulnerable patients and training them in their use could facilitate their utilization by the broadest possible population base. In order to meet patients’ needs and expectations these apps should be designed, developed, and improved in collaboration with a broad spectrum of patient partners. Currently, however, there are few regulations around mHealth apps and poor quality and incorrect information could mislead patients [35] and adversely affect their decisions and health outcomes. Developers of decision aid apps should apply international decision aid standards and quality control is a necessity, which requires manpower and a well-functioning regulatory climate [36].

Additional concerns include that some apps may also increase anxiety among patients. A recent study [37] showed that mHealth apps caused anxiety among breast cancer patients waiting for surgery by sending them multiple reminders. mHealth and medical apps may generate different risks to patients and their safety, and these risks could be even more harmful in complex apps [38]. There are also security concerns regarding patients’ information in mHealth apps, such as risk of data breaches or tampering [39]. Lastly, patients and healthcare professionals may require training to use these apps, involving further time and money investment.

## Conclusion

Nowadays mobile applications are accessible, affordable, and easy to use for patients and health providers in higher-income parts of the world, where in general there is increased interest in developing and using mHealth apps for supporting SDM. Recent studies suggest that mHealth apps can empower patients, encourage greater participation of patients in medical decision-making, and increase patient satisfaction. Other studies suggest certain potential disadvantages, such as increased level of anxiety of patients, limited access to Internet or mobile phone networks in lower-income countries, and security concerns.

Future studies should evaluate the extent to which current mobile apps that support SDM are consistent with agreed standards, such as the IPDAS. It is also important to find a way to measure whether mHealth apps advertised as decision support tools do in fact support SDM or not, and if they do support SDM, what their effects are on both patients and health providers. For mHealth apps to achieve the goals of SDM, patients should participate in the app development procedure. Moreover, there is a need for further research into understanding the impact of decision aid apps on the professional–patient relationship, how mHealth apps are presented to and received by patients, and on adapting them to a variety of cultural and socio-economic contexts. Finally, a systematic review of the evidence in this area is essential to identify and review mHealth apps designed to support SDM and informed decision making.

## Paper context

Mobile health applications are being developed to support SDM. However, in spite of increasing research on mHealth, few studies address mHealth in support of SDM. We discuss the potential advantages and disadvantages of using mHealth in shared decision making in a variety of contexts and suggest directions for future research in this quickly expanding field.
